# Cognitive development among children in a low-income setting: Cost-effectiveness analysis of a maternal nutrition education intervention in rural Uganda

**DOI:** 10.1371/journal.pone.0290379

**Published:** 2023-08-18

**Authors:** Montasir Ahmed, Grace K. M. Muhoozi, Prudence Atukunda, Ane C. Westerberg, Per O. Iversen, Knut R. Wangen

**Affiliations:** 1 Wolfson Institute of Population Health, Queen Mary University of London, England, United Kingdom; 2 Department of Health Management and Health Economics, University of Oslo, Oslo, Norway; 3 Department of Family Life and Consumer Studies (Home Economics), Kyambogo University, Kampala, Uganda; 4 Center for Crisis Psychology, Faculty of Psychology, University of Bergen, Bergen, Norway; 5 Institute of Health Sciences, Kristiania University College, Oslo, Norway; 6 Division of Obstetrics and Gynecology, Oslo University Hospital, Oslo, Norway; 7 Department of Nutrition, University of Oslo, Oslo, Norway; 8 Department of Haematology, Oslo University Hospital, Oslo, Norway; 9 Division of Human Nutrition, Stellenbosch University, Tygerberg, South Africa; The University of Jordan School of Pharmacy, JORDAN

## Abstract

Inadequate nutrition and insufficient stimulation in early childhood can lead to long-term deficits in cognitive and social development. Evidence for policy and decision-making regarding the cost of delivering nutrition education is lacking in low and middle-income countries (LMIC). In rural Uganda, we conducted a cluster-randomized controlled trial (RCT) examining the effect of a maternal nutrition education intervention on developmental outcomes among children aged 6–8 months. This intervention led to significantly improved cognitive scores when the children reached the age of 20–24 months. When considering the potential for this intervention’s future implementation, the desired effects should be weighed against the increased costs. This study therefore aimed to assess the cost-effectiveness of this education intervention compared with current practice. Health outcome data were based on the RCT. Cost data were initially identified by reviewing publications from the RCT, while more detailed information was obtained by interviewing researchers involved in processing the intervention. This study considered a healthcare provider perspective for an 18-months’ time horizon. The control group was considered as the current practice for the future large-scale implementation of this intervention. A cost-effectiveness analysis was performed, including calculations of incremental cost-effectiveness ratios (ICERs). In addition, uncertainty in the results was characterized using one-way and probabilistic sensitivity analyses. The ICER for the education intervention compared with current practice was USD ($) 16.50 per cognitive composite score gained, with an incremental cost of $265.79 and an incremental cognitive composite score of 16.11. The sensitivity analyses indicated the robustness of these results. The ICER was sensitive to changes in cognitive composite score and the cost of personnel. The education intervention can be considered cost-effective compared with the current practice. The outcome of this study, including the cost analysis, health outcome, cost-effectiveness, and sensitivity analysis, can be useful to inform policymakers and stakeholders about effective resource allocation processes in Uganda and possibly other LMIC.

## Introduction

Adequate nutrition is essential to lay the foundation for brain development [1]. Children younger than three years are especially vulnerable and dependent on their mothers for adequate nutrition and stimulation [2]. Nutrition education interventions affect child cognitive development in low and middle-income countries (LMIC). Specifically, there are positive associations between educational interventions and cognitive development during the first two years of life [3, 4]. Evidence also shows that child stimulation in the early years of life is associated with timely cognitive development [5, 6].

In Uganda, healthcare services are provided by the public and the private, not-for-profit sectors, also including faith-based Catholic, Protestant, and Muslim Medical Bureaus [7]. The nutritional status of many population groups is often poor, especially among children under the age of five years [8]. In line with this, a survey from 2016 showed that undernutrition is highly prevalent in rural Uganda [9], with 29% of children aged 6–59 months being stunted (i.e., low height-for-age and a marker for chronic undernutrition), 4% being wasted (i.e., low weight-for-height and a marker for acute undernutrition), and 11% being underweight [10]. Statistics show that there was an overall decline in wasting and stunting prevalence between 1995 and 2016; however, the annual reduction rate of stunting and wasting was only 0.45% and 0.01%, respectively [11]. Notably, only a few interventions targeting nutrition have been implemented in Uganda [12, 13].

To combat stunting, we conducted the “Child Nutrition and Development Study” (CHNUDEV) in 2013–14, a two-armed, pragmatic cluster-randomized controlled trial (RCT) among mother/child dyads in South-Western rural Uganda (www.med.uio.no/imb/english/research/projects/chnudev-study/index.html) [14]. In that RCT, we explored the effects of a maternal education intervention focusing on nutrition, hygiene and child stimulation, on child development. Data obtained from this RCT served as the data source for the current study’s analysis. Results from the CHNUDEV project have demonstrated limited effects on growth, but substantially improved cognitive, language, and motor development in the intervention group compared with the control group [14, 15]. The project has not yet presented cost and cost-effectiveness analyses, even though such analyses may form an important basis for deciding upon a large-scale implementation of the education intervention and is thus clearly relevant for stakeholders and policymakers.

Education interventions involving improvement of mother’s knowledge about nutrition and cognitive stimulation are known to be related to improved child health and survival, and cognitive development [16, 17]. Specifically, nutrition education interventions have been implemented in several countries, such as in rural Uganda [12], Bangladesh [18], China [19], and Peru [20], providing evidence of significantly higher cognitive scores in the intervention group compared with the control group. However, a significant policy challenge arises in terms of determining how nutrition intervention programs can be delivered at a large scale. Policymakers often require more precise information than is currently available to make early childhood development investment decisions [21]. Apart from the nutritional education intervention’s clinical effectiveness, its economic aspect also plays an important role in the decision-making process [22, 23].

We therefore aimed to assess the cost-effectiveness of the nutritional education intervention in comparison to the current practice, utilizing data from the CHNUDEV project. This analysis of cost-effectiveness was planned subsequent to the completion of the RCT and serves as an early health technology assessment. Our goal was to compare alternatives that would be relevant if local decision-makers were to contemplate expanding the educational intervention in the future.

Individual level health outcomes data were obtained directly from the RCT. Costs were initially identified by reviewing publications from the RCT, while more detailed information was obtained by meetings and interviews with RCT researchers and project leader. To the best of our knowledge, there have been no similar studies to assess the cost and cost-effectiveness of an education intervention to improve cognitive development among small children, either in Uganda or elsewhere.

## Material and methods

### Study setting and study participants

We conducted the original RCT in 2013–2014 in the Kabale and Kisoro districts of South-Western Uganda [14, 15]. The inhabitants in that region were mostly small-scale farmers cultivating small plots of land. Both districts were populated with individuals of similar socio-economic status and feeding practices, being densely populated, and having a high prevalence of stunting among children.

We recruited the mother/child dyads when the children were 6–8 months old (baseline), primarily because complementary feeding is generally recommended to start at 6 months of age, and since children of that age are among the most vulnerable to inadequate nutrient supply and poor linear growth. In total 511 mothers and children were enrolled into either an intervention group (n = 263) or a control group (n = 248) at baseline [15]. All mothers gave written or thumb-printed, informed consent to participate [15]. Details of the recruitment procedure and sample size calculation are given in S1 File.

### Intervention and comparator

The intervention aimed to promote behavioral change by providing prompt practice through access to information and improved application. It consisted of educating mothers to increase dietary diversity for improving nutrition intake, also emphasizing stimulation, sanitation, and hygiene [14, 15]. The intervention strategy included practical demonstrations in group sessions to educate and empower the mothers. Confer S1 File for further details.

The control group children received standard health care and were solely assessed in terms of their health outcomes. Their mothers did not receive any particular education intervention, as per the trial protocol.

Regarding the potential future implementation of this intervention, the relevant comparator will be the current practice, considering both health outcomes and costs. However, the health outcomes and costs associated with the current practice are unavailable. Consequently, our analysis should be considered as an early health technology assessment that relied on two assumptions: First, for the health outcomes, we assumed that the current practice will yield similar results to those observed in the control group during the trial. Second, for costs, we assumed that the costs of the intervention will be in addition to the costs associated with the current practice.

### Health outcome: Child development

The health outcome for the current study was cognitive development, measured by the cognitive composite score according to the Bayley Scales of Infant and Toddler Development-III (BSID-III), on the subscales of cognitive, language, and motor development [24]. The raw cognitive scores were converted to composite scores according to BSID-III conversion tables [14]. The BSID-III scales were adapted for usability relevant to the cultural context of the study population and translated back to English. Trained field-teams independently collected the data. Child assessments were performed in a hired special room to avoid interference. The study personnel assessing the children were blinded to group allocation.

In addition to cognitive development, the CHNUDEV project also measured other developmental outcomes, such as language and motor development, communication, problem-solving, and personal and social development. It is worth noting that cognitive development is known to be interconnected with motor development [25, 26] and is influenced by language development [27]. As a result, cognitive development is linked to the broader spectrum of health outcomes. However, for the purposes of this study, cognitive development was considered the sole health outcome for analysis.

### Data collection

We defined the pathway of how the education intervention was delivered to assess the health outcome. At the beginning, some costs were identified from the review of the published literature from the RCT [14, 15, 28]. Later, we collected cost-related data by conducting a meeting with the RCT researchers and project leader. Cost data were sought via a telephone interview with one clinical psychologist, one nutritionist, and one professor of clinical nutrition at the University of Oslo. These scholars were chosen for their expertise in planning and executing throughout the RCT. The authors MA and KRW estimated the costs via review of internal documents and project records, while the health outcomes were estimated based on data from the RCT.

### Cost estimates

Costs were assessed from a health care provider perspective. Costs were estimated using 2014 figures and we denote US dollars as $ (2014 USD 1.00 = 2,523 Uganda Shillings). To define precise cost specifications, we followed a few other similar studies [29–32], and the ISPOR recommended guidelines [33]. Costs were classified according to major expenditure lines to understand the depth of the RCT implementation and its associated costs. The costing was done across four key categories which were identified as the main activities of the education intervention. Those included personnel, materials and other costs, capacity building, and capital costs. Cost categories, source, number of units, unit price, and item costs are available in S2 File.

#### Capacity building

Capacity building comprised training and a follow-up study, with the essential task involving recruitment and training of health workers to implement the intervention. It included providing information and promoting practices such as demonstrations of preparing food and stimulation of the children [14]. The number of participants in the training session and unit costs are available in S2 File.

#### Personnel cost

Cost-wise, health workers were the most significant element required to implement this education intervention. Full-time personnel, part-time employees, and health workers were hired to conduct the intervention. The management team supporting the intervention was led by a clinical psychologist and supportive supervision composed of nutritionists who were trained in administering the child development tools and scoring the performance of the children. The details of personnel categories, responsibilities, and salary are available in S2 File.

#### Materials and equipment costs

Most of the materials and other costs were equally shared between the two study groups. For instance, the team’s transportation costs, data collection materials, incentives (t-shirt) and refreshment costs, were shared equally between the intervention and control groups of mothers and children. Scales, tapes, length boards, and picture booklets were only used during consultancy sessions, while a few specific materials were solely allocated to the intervention group, such as the nutrition intervention demonstration, adherence to intervention, and facilitation of intervention. The food demonstration had a purchased unit cost of $14.50, and we performed 150 nutrition education sessions for mothers. Other costs referred to those that did not fit readily into the categories set out above, for instance, the transportation, refreshment, and incentive costs. All the participants’ mothers received a t-shirt as an incentive for taking part in the study.

#### Facilities and other inputs

The proportion of facilities and other inputs spent on this intervention was low, accounting for around 1% of the total cost. Child assessments were performed in hired special rooms in the villages, while a mobile tent was used when such rooms were not available. A mobile tent had a fixed cost, while the rented room had a monthly expenditure, and both places accommodated baseline and follow-up assessments for the respective mothers and children. The RCT team members used three mobile phones throughout the study period.

### Cost-effectiveness assessment criteria

This study used the cost and health effect of the RCT endpoints, when the children were 20–24 months old, following the ISPOR guidelines [33]. The intervention costs were estimated by calculating the average cost for the intervention group, assuming that these costs represented an additional expense compared to the unobserved costs associated with the current practice. Therefore, the incremental costs were determined as the difference between the estimated average cost for the intervention group and the assumed cost for the comparator (i.e., zero). The costs of the control group in the RCT were not utilized in the cost-effectiveness analysis as they were specific to the trial protocol and lacked relevance in the context of current practice. The health effect of the intervention was measured using the mean cognitive composite score of the intervention group in the RCT. As for the comparator, current practice, the health effect was defined as equal to the mean cognitive composite score of the control group. The incremental health effect was calculated as the difference in mean cognitive composite scores between the intervention group and the comparator (i.e., the cognitive composite score for the control group). The incremental cost-effectiveness ratio (ICER) was defined as the ratio between the incremental cost and the incremental health effect. Since the study period was relatively short (18 months), corresponding to our time horizon, we did not apply discounting to account for differential timing of costs and effects. Given that the time horizon in the cost-effectiveness analysis was only 18 months (corresponding to the study period), we did not use discounting to account for differential timing of costs and effects.

To the best of our knowledge, no link has been established between generic measures of health effects, such as avoided Disability Adjusted Life Years (DALY), and our health measure (i.e., units of cognitive composite score). However, based on the World Health Organization’s generally suggested willingness to pay for avoiding the loss of a DALY, we performed approximated calculations that could help relate our results to previously published DALY weights for mild cognitive impairments (see the and the Discussion)

### Statistical analyses

Chi-square tests were used in analyses for categorical variables. Two-sample t-tests were used for numerical variables. A p-value of <0.05 was considered significant. We obtained an estimate of the incremental composite cognitive score through a mixed model linear regression. In this regression, the cognitive score of each individual (the dependent variable) was observed at baseline and at the last follow-up. The regression model included a random intercept at the village level and a random intercept at the individual level. Additionally, all individual baseline characteristics from Table 1, except language development and motor development (i.e., development measures that partly overlap with the dependent variable), were included at the individual level. The incremental cognitive composite score was measured as the coefficient of the interaction between two dichotomous variables: group (control = 0, intervention = 1) and time (baseline = 0, follow-up = 1). Stata/SE version 17.0 was used for statistical analyses. Microsoft Excel was used to perform the cost effectiveness analyses.

**Table 1:** Demographic and dietary characteristics of mothers and children at baseline.

Variable description	Intervention	Control group	P-value
	group (n = 263)	(n = 248)	
**Categorical variables**	**Number (percentage)**		
Child gender			0.462
Male	139 (52.9)	123 (49.6)	
Female	124 (47.2)	125 (50.4)	
Breastfeeding frequency			0.032
Breastfeeding on demand	170 (64.9)	172 (73.8)	
Breastfeeding < = 8 times a day	92 (35.1)	61 (26.2)	
Started complementary feeding			0.420
Yes	254 (96.6)	236 (95.2)	
No	9 (3.4)	12 (4.8)	
Dietary diversity score			0.015
Low dietary diversity	149 (56.7)	168 (67.7)	
Medium dietary diversity	83 (31.6)	65 (26.2)	
High dietary diversity	31 (11.8)	15 (6.0)	
**Numerical variables**	**Mean (standard deviation)**		
Child age at inclusion, in months	7.39 (0.83)	7.26 (0.91)	0.095
Weight-for-age, z-score	-.63 (1.10)	-.72 (1.13)	0.343
Weight-for-length, z-score	0.12 (1.21)	0.15 (1.26)	0.813
Length-for-age, z-score	-1.07 (1.15)	-1.21 (1.24)	0.214
Head circumference z-score	.68 (1.08)	.57 (1.18)	0.254
Cognitive composite score, BSID-III	101.99 (12.84)	103.52 (13.85)	0.222
Language development, BSID-III	103.57 (14.35)	100.13 (14.01)	0.010
Motor development, BSID-III	104.82 (13.73)	104.40 (14.68)	0.757
Maternal education, in years	4.85 (2.82)	4.92 (2.75)	0.781
Maternal age, in years	26.17 (5.92)	26.98 (6.5)	0.154
Number of children per mother	3.43 (2.24)	3.34 (2.23)	0.674
Mother age during first child	19.57 (2.61)	19.61 (3.02)	0.891
Household head age, in years	31.36 (7.90)	33.06 (10.74)	0.048
Household head education, in years	6.38 (3.13)	5.91 (3.05)	0.093
Household size	5.47 (2.08)	5.48 (2.09)	0.965
Household poverty score	47.84 (11.65)	47.61 (11.38)	0.824
Likelihood to be below poverty line	14.57 (15.91)	15.05 (15.71)	0.735

The p-values are from chi-squared tests for the categorical variables, and t-tests for the numerical variables. BSID-III: Bayley Scales of Infant and Toddler Development-III.

### Sensitivity analyses

The influence of parameter uncertainty on the results was assessed through a one-way sensitivity analysis and presented in a tornado diagram. The incremental cognitive composite score was varied according to the upper and lower limits of the 95% confidence interval obtained from the regression. The personnel cost varied from being 50% reduced to being 20% increased. The low value was decided on the assumption that the cost of personnel would gradually be decreasing with the increasing number of participants. Concerning materials and other costs, we applied a range of ±20% from the baseline price. This could be explained by the fact that if the number of participants increased, the cost of materials and other costs would also increase. It is noteworthy that capital costs were around 1% of the total costs. Thus, the same range of ±20% was applied to test sensitivity analysis for the capital cost. The list of parameters, point estimates in deterministic and probabilistic sensitivity analyses are available in S2 File.

A probabilistic sensitivity analysis (PSA) was performed using Monte Carlo simulation, in which distributions were assigned to the uncertain model parameters. For the incremental cognitive composite score, a normal distribution was used with the mean and standard deviation set equal to the point estimate from the regression and its standard error, respectively. As for the cost parameters, uniform distributions were used, with ranges corresponding to those employed in the one-way sensitivity analysis. Assuming the parameters were independently distributed, we drew 1000 samples. We described these samples by creating a scatter plot that visualizes the joint distribution of incremental costs and incremental benefits. Additionally, we generated a cost-effectiveness acceptability curve to compare the intervention with the current practice.

### Approvals

This RCT was approved by the Uganda National Council for Science (UNCST) Ref. HS 1425, having been reviewed by the Makerere University School of Public Health, Higher Degrees Research and Ethics Committee (no. IRB000353), and the Norwegian Regional Committee for Medical and Health Research Ethics (no. 2013/1833), and it was registered in ClinicalTrials.gov ID NCT 02098031.

## Results

### Background information of the participants

Table 1 presents the baseline characteristics for the two study groups. The mean child age in the intervention and control group was 7.39 and 7.26 months, respectively. Apart from breastfeeding frequency, language development, and household head age, all the variables had a p-value>0.05. Thus, the baseline characteristics and demographic variables seemed well balanced between the two study groups.

#### Effectiveness—the cognitive composite score gained during follow-up

In the RCT, the number of participants differed from the baseline to the last follow-up. When calculating the cognitive composite scores in the cost-effectiveness analysis, we retained those children who were assessed at the baseline and then continued throughout the last follow-up, respectively 227 and 196 children in the intervention and control groups (423 children in total).

**Fig 1:**
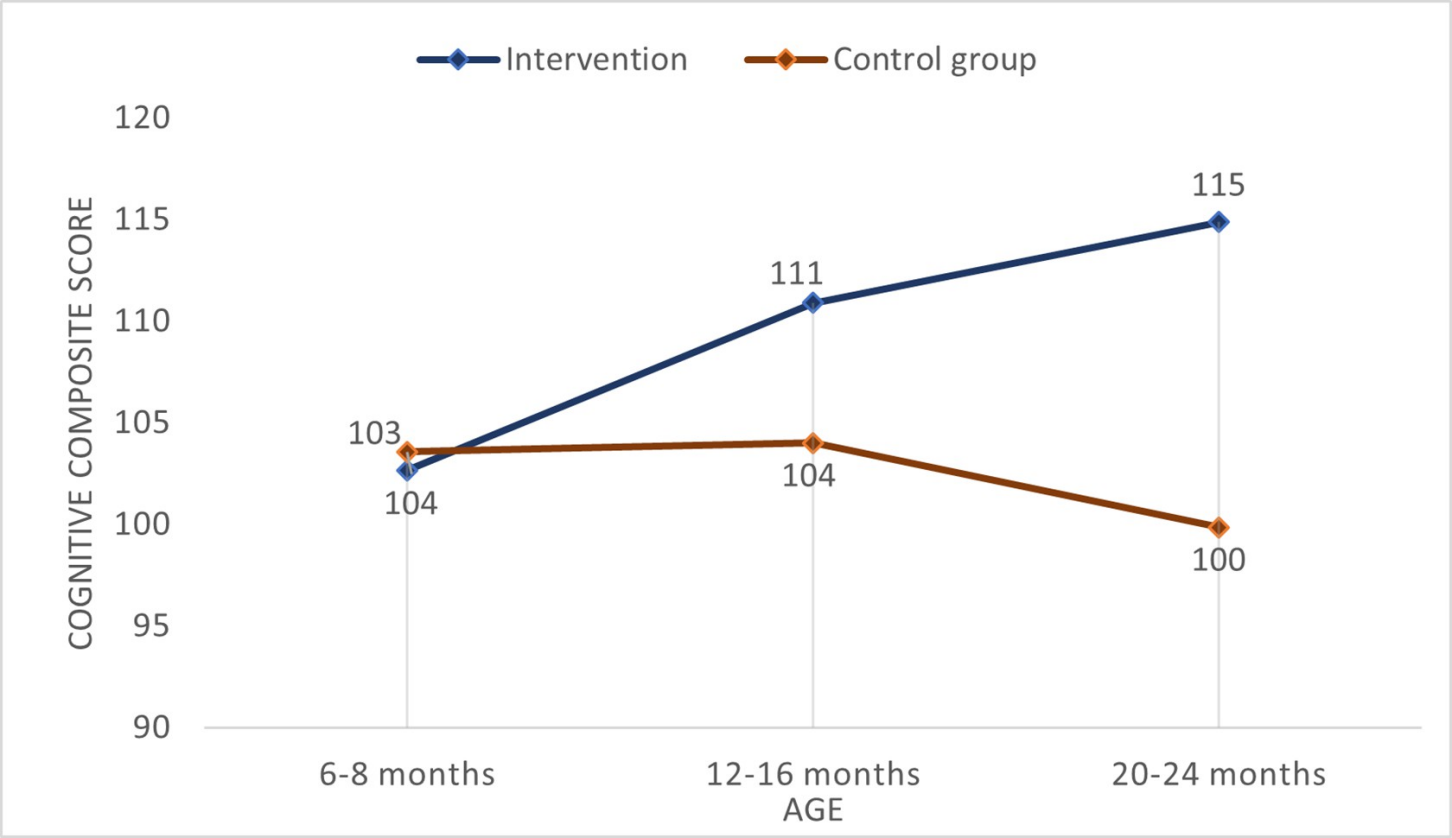
Mean cognitive composite scores at baseline and follow-up.

Fig 1 illustrates the mean cognitive composite score at baseline and the two follow-ups. At baseline, the mean cognitive composite scores for the control group and the intervention group were, respectively, 103.42 and 102.37, and the difference was not significant (p = 0.425). At the age of 12–16 months, there was a trend toward improvement of mean cognitive composite score in the intervention group (110.50) compared with the controls (103.31), which was a significant difference (p = 0.000). Finally, at the last follow-up when the children were aged 20–24 months, children in the intervention group had, on average, 15.30 units higher cognitive composite score than those assigned to the control group (114.67 versus 99.37; p = 0.000).

The estimate of the incremental composite cognitive score from the mixed model linear regression was 16.11 (95% confidence interval: 11.72 to 20.50). This estimate is slightly higher than the difference between the means observed at the last follow-up (Fig 1). This discrepancy can be attributed partly to the intervention and control groups having slightly different mean scores at baseline, and partly to the influence of the individual baseline characteristics and random intercepts on the regression estimate. None of the individual baseline characteristics were found to be statistically significant in the regression.

### Costs

The total intervention costs amounted to $60,335, resulting in an average cost per child of $265.79 when divided by the number of children in the intervention group (n = 227). Personnel costs accounted for the largest proportion, representing 77% ($46,485). Materials and other variable costs accounted for 19% ($11,496), followed by capacity building costs at 3% ($1,822), and other overhead/fixed/capital costs at approximately 1% ($533). Further details of the intervention costs are provided in S2 File (S1 Table in S2 File).

### Cost-effectiveness

The deterministic cost-effectiveness results are summarized in Table 2. The average intervention cost was $265.79 per child in the intervention group. Given our assumption that these costs represented an additional expense compared to the unobserved costs of current practice, this value represents the incremental costs. The incremental (health effect) cognitive composite score was obtained from the mixed model linear regression and represents the intervention’s average health benefit per child. The resulting ICER for the education intervention compared with current practices, which is the ratio of incremental costs and incremental health effect, was $16.50 per cognitive composite score gained.

**Table 2:** Cost-effectiveness result for the intervention versus current practices at 20–24 months.

Incremental cost ($)	Incremental cognitive composite score	ICER
265.79	16.11	16.50

Costs were estimated using 2014 figures in USD ($). The incremental cost effectiveness ratio (ICER) is measured in $ per cognitive composite score.

### Sensitivity analyses

**Fig 2:**
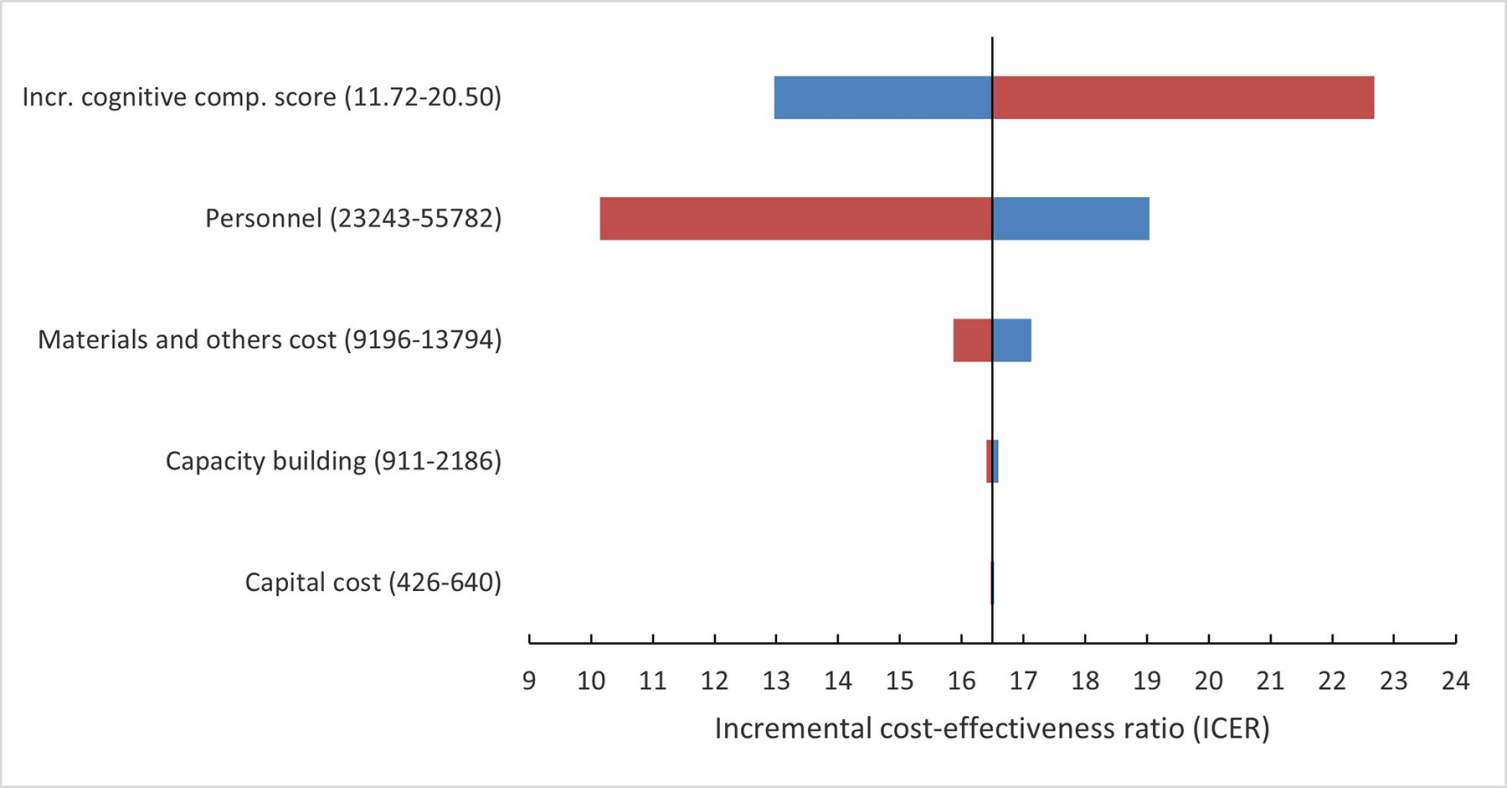
Tornado diagram: One-way sensitivity analysis of parameters’ effect on the ICER. The ICER is measured in $ per cognitive composite score. A blue bar represents the effect on the ICER of increasing a parameter value from its reference value, while a red bar represents the effect of reducing a parameter value. All increases and reductions in the graph represent a 20% change from their respective reference parameter values, except for personnel cost where the red bar represents a 50% reduction, and the incremental cognitive composite score where the parameter values were set to the limits in the 95% confidence interval.

The key parameters were cost of personnel, materials and other costs, capacity building, and cognitive composite score, which were found to have a significant impact on the ICER. The results of the cost categories and cognitive composite score were combined into a tornado diagram and presented in Fig 2. The tornado diagram shows that the ICERs range between $10.14 and $22.68. The impact of incremental cognitive composite score on the ICERs is not linear. Lowering the score from 20.50 to 11.72 resulted in an increace in the ICER from $12.97 to $22.68. When the personnel cost was reduced by 50%, the ICER decreased to $10.14. Conversely, when personnel costs increased by 20%, the ICER increased to $19.04. Thus, both incremental cognitive composite score and cost of personnel had the greatest effect on the ICER. Varying the other parameters had little impact on cost-effectiveness results.

**Fig 3:**
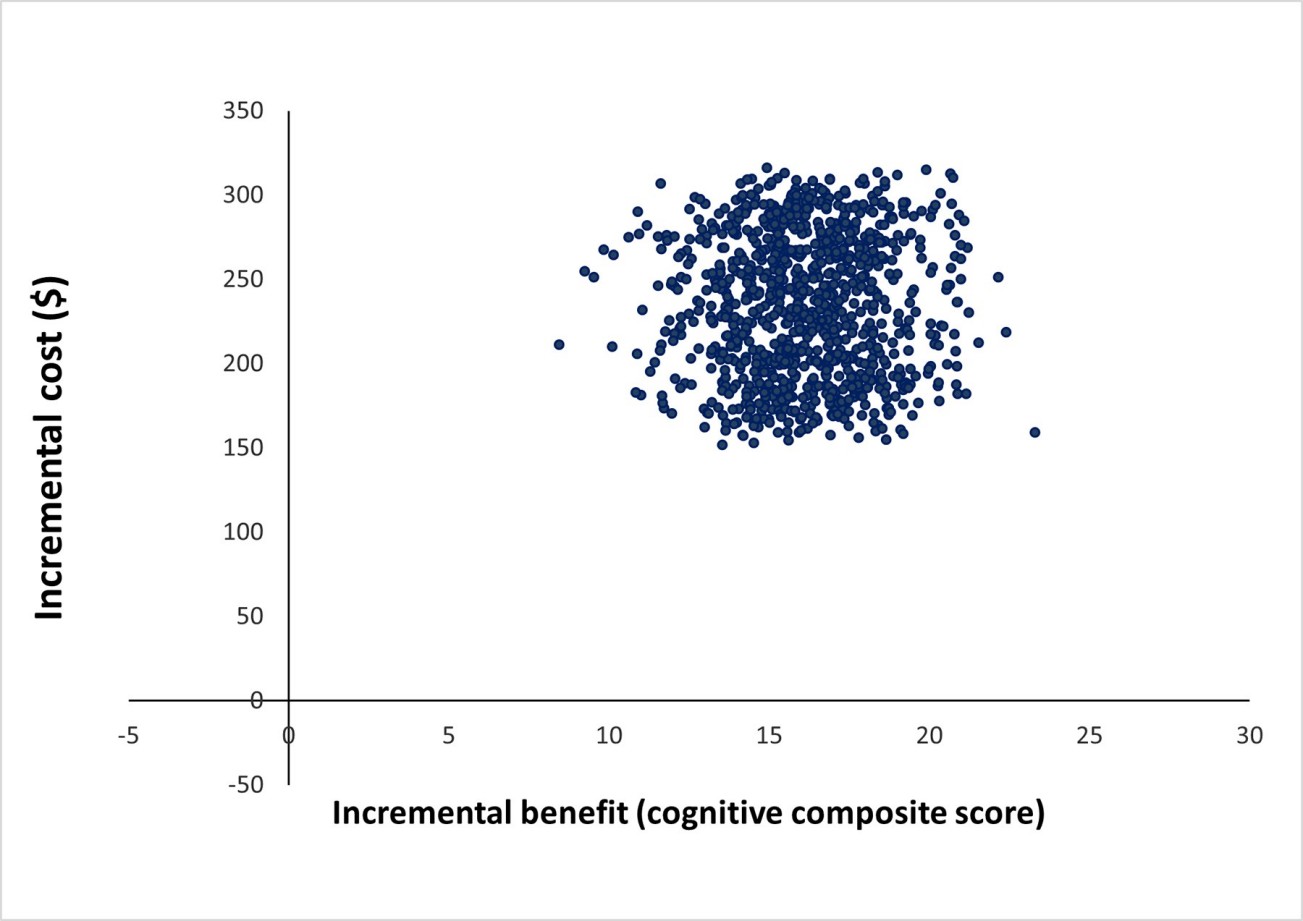
Scatter plot of the joint distribution of the incremental cost and the incremental benefit of the nutrition education intervention compared to the current practice Each dot represents one out of 1,000 Monte Carlo replications. The distributions used in the simulation are specified in Supplement 2, S3 Table in S2 File.

Fig 3 presents the results of the probabilistic sensitivity analysis on the cost-effectiveness plane. Each point represents the incremental cost and incremental cognitive composite score for one of the 1000 Monte Carlo samples. All the data points are situated in the top right quadrant, indicating that the education intervention is both more costly and more effective than the current practice.

**Fig 4:**
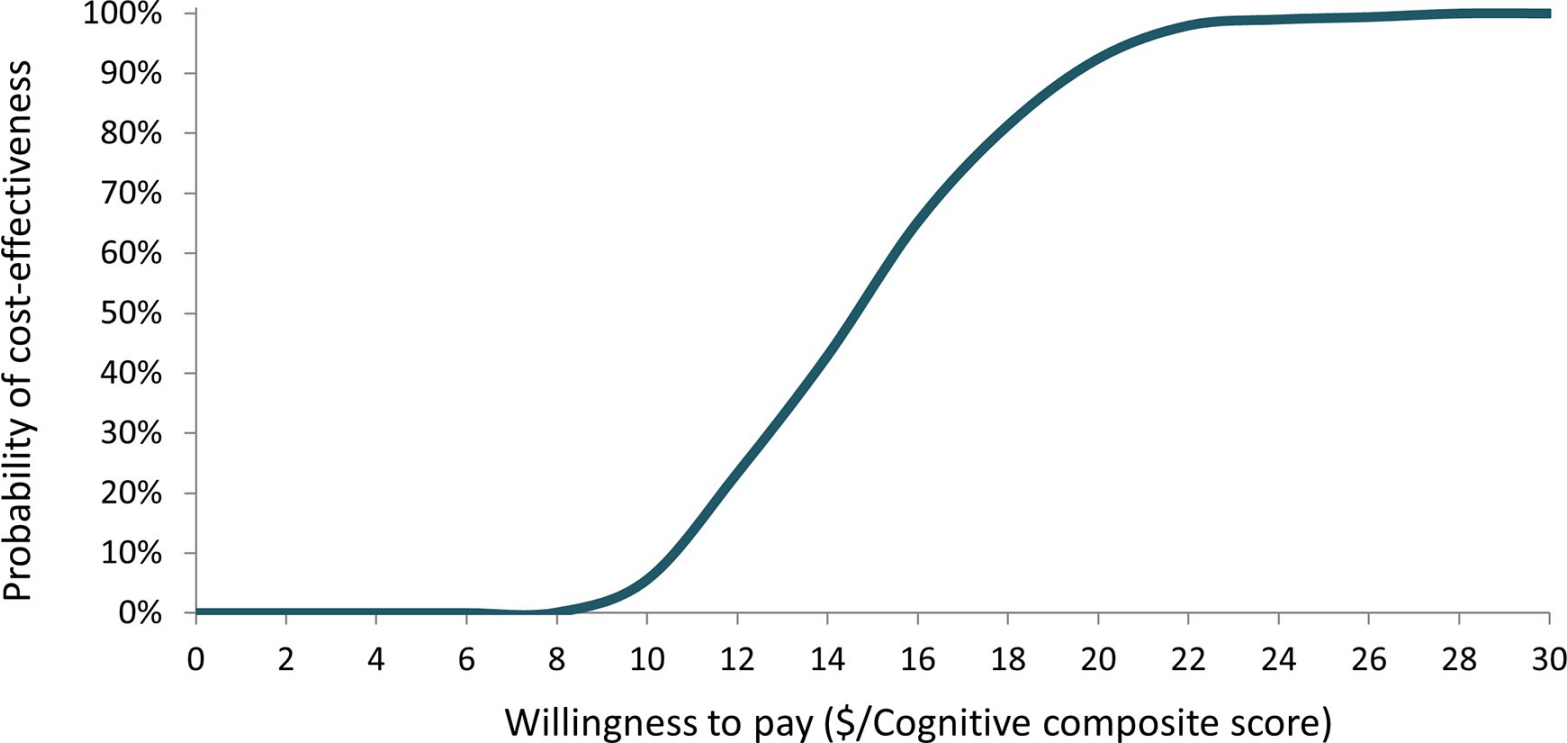
Cost-effectiveness acceptability curve of the nutrition education intervention versus the current practice.

Finally, we constructed a cost-effectiveness acceptability curve (CEAC) to assess the likelihood of the education intervention being cost-effective across different willingness to pay thresholds for an additional cognitive composite score gained. Fig 4 illustrates the probabilities at various thresholds. At a willingness to pay threshold of $8, the intervention had an approximate 0% probability of being cost-effective. At the deterministic willingness to pay threshold of $16.50 per unit of cognitive composite score (as shown in Table 2), the education intervention had increased to an approximate 71% probability of being considered cost-effective. The probabilities indicated by the CEAC were nearly 100% when the threshold was set at $24.

## Discussion

In Uganda, the Ministry of Health assumes leadership and is responsible for resource mobilization, operating under the following hierarchical structure: National referral hospitals, Regional referral hospitals, Health center IV at the Sub-district level, Health center III, Health center II, and the Village Health Teams.

Planning and decision-making are integral responsibilities of the District Health Management teams. However, the decision-making process is ad hoc and dependent on the availability of funds [34]. The majority of funding is obtained from donors and partners who have their specific priority areas. Consequently, at lower levels, such as where our research was conducted, essential health services and supplies that are urgently required are not promptly delivered in sufficient quantities. As a result, a significant portion of the population is deprived of these services, with only a limited few able to afford them.

The main objective of this cost effectiveness analysis was to aid potential decision-makers and stake-holders by providing an economic evaluation of health outcomes and costs. This study considered important concerns with respect to a future large-scale implementation of the education intervention. One significant aspect was that this education intervention was largely managed and implemented by local personnel, using local village health teams. Thus, the cost-effectiveness findings of this intervention would have the potential to be replicated in other low-resource, community-based settings.

Health promotion for parenting programs has consistently been linked with early childhood development and cognitive development [12, 35–38], and studies conducted in 40 LMICs found that early childhood interventions can have a reliable and positive effect on cognitive development [39]. Although the evidence base for the importance of educational interventions for cognitive development has grown, there is less agreement about the most effective and efficient way to enhance cognitive development. Economic evaluations have rarely been conducted on this topic in LMICs [21], and there are generally few national statistics available on the development of young children in LMICs [40].

Our results show that the mean cognitive composite score increased with 16.11 units in the intervention group relative to the control group. Similar cost-effectiveness analyses of education interventions delivered to mothers to enhance children’s cognitive development are rare and we have only found one, which was conducted in Pakistan. That study reported that the cognitive composite score difference between the group with responsive stimulation and that without responsive stimulation was 7.6 (the average scores were 81.7 and 74.1, respectively) [41].

Our estimated ICER indicates that the education intervention would be a cost-effective strategy if the willingness to pay threshold is above $16.50 per cognitive composite score gained. However, it has not been stated an explicit official willingness to pay threshold for cognitive development in Uganda. When used to guide high-level decision makers, cost-effectiveness analyses preferably include generic health measures such as DALY. In our study, it was not possible to translate the cognitive composite score to DALYs because both the DALY weight and the duration of the health effect were unknown. However, we present numerical examples in the S2 File, which show that the intervention could be cost effective for an average gain in cognitive composite score (16.11 units), if the corresponding, but unknown DALY weight is >0.002 (assuming a 3-GDP-per-capita willingness to pay per DALY), and very cost effective if the DALY weight is >0.005 (assuming a 1-GDP-per capita willingness to pay per DALY) [42]. Albeit it was beyond our scope to estimate the DALY weight, we note that the DALY weight for the condition “Mild Motor and Cognitive Impairment” has been reported to be 0.031 [43].

In the probabilistic sensitivity analysis, the estimated mean ICER was $14.61 (95% decision model interval $9.14-$21.48) per unit of cognitive composite score, and all individual simulations were distributed in the top right quadrant. The deterministic one-way sensitivity analysis (Fig 2) revealed that the input parameters for cognitive composite scores and the personnel costs had clearly the most influence on the ICER. Lower cognitive composite scores would lead to a higher ICER and tend to make the intervention less favorable. This finding highlights an important message for a future implementation of this intervention: training of personnel and supervision should be highly prioritized to maintain the education intervention quality.

High personnel costs are common in educational intervention programs and tend to decrease marginally over time with increasing number of participants [30]. We find it likely that the original RCT will have higher personnel cost in comparison with a future implementation of the education intervention also because the RCT assessed more health outcomes. For instance, while the personnel spent 1.5 hours on average to assess individual child health outcomes, the BSID-III scale involved an average of 40–60 minutes to assess child development outcome [24]. Our available data on the personnel’s working hours were not sufficiently detailed to identify time spent on each type of task. However, if the personnel costs could be reduced by 50%, which does not seem unreasonable, the ICER would be reduced to around $10 per cognitive composite score gained (Fig 2, lower bound for Personnel costs).

Relative to personnel costs, the remaining cost categories had minor impact on the ICER (Fig 2). However, in scaling up the education intervention, attention should be given to how the average cost could be affected by the increasing number of participants. Most of the materials and other cost were variable costs that would increase nearly proportional with the number of participants. This includes incentive costs, such as money spent on t-shirts, which presumably can be kept approximately constant per participant so that the average cost per participant will not be affected. In the RCT, each child required a BSID-III test kit for each assessment of the cognitive composite score, so that also these costs can be kept nearly constant per participant. However, the purchase of larger quantities of t-shirts and test kits could potentially reduce the unit prices (volume discounts) and, hence, reduce the average cost per participant. Capacity building costs and capital costs were mostly one-time costs in the RCT, and in a future scaling up it is likely that these cost categories would increase less than proportionally with the number of participants.

One main strength of this study is that the analysis was based on a cluster-RCT; a rigorous study design that reduces selection bias [44]. A limitation of this study could be the accuracy of the cost data, as these were obtained via interviews with the researchers, rather than observing detailed expenditure records. An ideal practice for a cost-effectiveness analysis is to collect detailed cost data concomitant with the RCT [33], whereas our cost-effectiveness analysis was planned after conducting the RCT [30]. Another limitation is the restrictions implied by taking the healthcare provider’s perspective. The early childhood is a critical period for cognitive development, having vital effects on schooling, labor market outcomes, and spillover effects on other health outcomes [18, 41, 45]. Such aspects could, in principle, have been considered by taking a societal perspective but that would require the availability of long-term data.

## Conclusion

Education intervention compared with current practice might be considered cost effective in improving cognitive development for small children in rural Uganda, that is, money spent today will serve to enhance cognitive development in the future. The results of our sensitivity analyses revealed that the gain in cognitive composite score and the cost of personnel had a large impact on the ICER. The outcome of this analysis, including the cost, health outcome, cost-effectiveness, and sensitivity analysis, can be a useful tool to inform policymakers when resource allocations are prioritized.

There is still a knowledge gap to be filled in the area of education intervention and child health. The current study only considered the health outcome of the education intervention for about 18 months. Economic evaluations based on randomized trials are usually followed up for a shorter period, rarely considering a lifetime time horizon. Further research should consider a longer timeframe for the cost-effectiveness analysis, to provide relevant and more reliable empirical evidence of the education intervention.

## Supporting information

S1 File Description of the original cluster-randomized controlled trial. (DOCX)

S2 File Supplementary methodology for the economic evaluation. (DOCX)
