# Supplementary material for: Cognitive development among children in a low-income setting: Cost-effectiveness analysis of a maternal nutrition education intervention in rural Uganda
Source: PLoS One. 2023 Aug 18;18(8):e0290379. doi: 10.1371/journal.pone.0290379 (PMC10437995; doi:10.1371/journal.pone.0290379)
Supplement: S1 File — (DOCX) [file pone.0290379.s001.docx]

**S1 Supporting information file**

**Description of the original cluster-randomized controlled trial**

**Recruitment and sample size calculation**

A simple random sampling was performed to allocate 10 sub-counties (clusters) in each district (6 from Kabale and 4 from Kisoro districts) to either the intervention or control group. All villages in each sub-county (intervention or control) were listed alphabetically and computer-generated random numbers were then used to obtain the villages, and finally complete enumeration was used to obtain participating households. Intervention villages did not share common geographical boundaries with control villages to prevent “contamination” of intervention-contents between the two study groups. Exclusion criteria were congenital malformations or physical handicap among children that would influence food intake, growth, mental or brain illness as evidenced by mother or health worker.

In the original cluster-randomized controlled trial, the sample size calculation was based on the primary outcome which was height-for-age z-score (HAZ) at child age of 20–24 months. The mean ± SD for HAZ is 0.0 ± 1.0 in a healthy population. We defined a difference of 0.3 SD in HAZ between the intervention and control group as clinically relevant, corresponding to about half a percentile in HAZ [1]. To detect a change of 0.3 SD in HAZ with a significance level of 5% and a power of 80%, 176 children were required per group. Fifty‐one children per sub-county were included presuming 10 sub-counties as clusters and an intra-cluster correlation of 0.01 [2]. To also account for dropouts etc., we ended up by including 511 mother/child dyads, and the assessment was by intention-to-treat.

**The education intervention**

An education intervention emphasizing nutrition, hygiene, sanitation and stimulation was delivered to mothers in the intervention group. Cooking and oral hygiene demonstrations together with making of play toys to promote child stimulation were parts of the education intervention package. The intervention lasted six months in which each group of mothers received three main education sessions (with a nutrition education team) followed by monthly mother group meetings to remind them of key aspects of the intervention. Our strategy with the intervention was to promote behaviour change through providing information and prompt practice (demonstrations). The intervention is detailed below.

**Nutrition education**

The nutrition package was centred on PAHO/WHO guiding principles of complementary feeding of a breastfed infant (quality and quantity of complementary feeds) [3]. The main emphasis was on:

- The importance of breastfeeding and a demonstration of how to position and attach the infant to the breast.
- The need to allow emptying one breast before changing to the other breast so that the infant could benefit from both the fore and hind breast milk.
- Breastfeeding eight or more times in a day including at night.
- All mothers were asked to start complementary feeding if they had not done so, since all infants were between 6 and 8 eight months of age.
- In complementary feeding, they were advised to start with soft foods in small amounts at a time and gradually increase the portion and the thickness of the food.
- Providing food that is rich in variety of nutrients and the importance of combining a variety of foods in one dish.
- To give infants complementary foods 2-3 times a day and increase the frequency of feeding to 3-4 times a day as the child grew.
- Providing nutritious healthy snacks (such as fruit) to the infant in between the main meals.
- Interaction and responsiveness while feeding the infants by talking, smiling and
  encouraging them to eat more without forcing them; to exercise patience and make
  feeding session a time for joy and bonding.
- To allow the infants to eat finger foods which they could hold with their hands.
- Continued breastfeeding until the child was 24 months of age.
- Breastfeeding more frequently, providing more fluids during illness (especially in diarrhoea and fever) of the infant, and giving foods that are more nutritious after recovery.

**Cookery demonstrations**

The cookery activities involved:

- Dishes which could combine up to 13 different foods in one obtained in their local
  environments.
- Inexpensive formulated recipes using locally available foods with emphasis on animal protein obtained from silverfish (*Rastrineobola argentea)* locally known as *Mukene.*
- Soy milk making, scraping meat (muscle), preparation of pumpkin seed powder and silverfish powder to incorporate in the infant’s food, addition of oil/fat and sugar to
  porridges to increase the energy content.
- Preparation of enriched porridge recipe 1 and 2 which were enriched with the
  ingredients of; cooking oil, sugar, silver fish powder, milk, pumpkin seed
  powder and eggs; in combinations of two or more.
- Preparation of scrambled eggs preferred to the boiled eggs or omelette, which are
  rather hard for the infants to consume.

**Hygiene education**

Themes of emphasis included:

- The importance of living in a clean home environment for the good health of the
  family particularly the young children.
- The basic requirement to always wash hands and utensils with clean water and soap during food preparation and infant feeding.
- The prerequisite to clean food before preparation to make it free of soil and other
  contaminants.
- Mothers were encouraged to carry water and a piece of soap to the field/gardens to wash hands before feeding the infants.
- Mothers were warned on giving leftover foods to the infants, since safety of such food was not possible and safe for the infants to consume later.
- Licking spoons as they fed the babies (to test the temperature) was discouraged to avoid transmission of infections from the mother to the infant.
- Oral hygiene was emphasized, which included cleaning the mouth of the baby with a clean cloth and warm water. Children and the entire family were later given tooth brushes with emphasis on how to use, clean and store them.

**Child stimulation**

The child play and stimulation emphasized:

- The importance of age graded child play activities and the role of mothers, other family members to engage in child stimulation.
- The significance of play to promote healthy development of the child.
- Explanation of the three development domains (cognitive, language and motor
  domains).

We explained to the mothers that the aim of play was to develop imagination creativity and social skills in the child [4]. The mothers were encouraged to use “name and identify” child’s body parts to facilitate the child’s understanding during his/her daily routine related to his body [5]. Practically, mothers engaged children in some of child play activities such as hiding favourite items for children to find; screwing and unscrewing bottles and imaginary play. Mothers also hand-made “easy to make” toys (from local materials) which were recommended as appropriate for children; shakers, empty transparent bottles with screws and food pellets inside, baby dolls made from cloth or banana fibres.

Language development was defined as verbal and non-verbal communication (expressive and receptive language) [6]. “We Talk” slogan was used to show mothers the importance of talking to the child so that they learn to talk back and, in the process develop language skills [6]. Mothers were encouraged using communication development aides such as imitation, roleplaying games, songs and music, to facilitate the child’s ability to communicate emotions, thoughts, needs and interests [7,8]. The mothers were encouraged to set aside time to purposefully talk to the children, call them by their name and to respond to them in word and/by gesturing; mention household and personal items while pointing at them, naming domestic animals, imitating their words and actions.

For motor development, the “Learn whereas playing” slogan was emphasized. The concept of gross motor skills was explained as the use of coordination and control of the body to facilitate the development of security, speed, and accuracy [9] in daily performance of tasks in a child’s life (larger movements like walking and kicking). Fine motor skills were defined as the ability to perform complex skills for more proficient tasks of daily living [7] (smaller movements like writing, tying shoelaces, and unbuttoning clothes). The following activities were emphasized:

- Giving child items to hold with their fingers, for example handing a pencil and paper for them to scribble.
- Matching lids with same size colour and shape games.
- Threading with beads
- Poking straws into holes.
- Stacking cups

The recommended toys included balls, bottle lids, cups, big beads, threads, ropes, shakers, pencils and paper. Furthermore, the mothers were encouraged to empower each other, by meeting regularly in their groups to practice and evaluate their childcare skills. We also advised them to be active with their sub-county activities for easy identification by government programs targeting women.

**Booster sessions of the educational components after the intervention period**

To prolong the effects of, and adherence to, the education intervention after the 6-months’ intervention period had ended and until the children were aged 20-24 months, we administered booster sessions to groups of 6-12 women in the intervention arm. These sessions (each lasting about 6 hours) were provided by the education team every third month and started three months after end of the intervention period, hence a maximum of 4 booster sessions were given for each group. The sessions were reminders of the education activities taught during the intervention period and re-emphasized the importance of (i) making nutritious meals; (ii) hand-washing and hygienic preparations, and (iii) child stimulation.

**Routine health care practices**

The intervention group received routine health care and the education intervention while the control group received only routine health care. The routine health care consisted of the recommended regular anthropometric measurements, immunizations, deworming, vitamin A supplementation, malaria-prophylaxis and iron-deficiency anemia prevention. Importantly, when the children were aged 20-24 months, we found that mothers in the intervention group had gained significantly more knowledge and better practices related to child feeding, hygiene and stimulation [10] compared to the control mothers, indicating that the contents of our education intervention differed markedly from routine health care.

**References**

1. Ong, K. K., et al., Association between postnatal catch‐up growth and obesity in childhood: Prospective cohort study. BMJ, 2000;(320): 967–971.
2. Campbell, M. J., et al., Developments in cluster randomized trials and Statistics in Medicine*.* Statistics in Medicine, 2007;(26): 2–19.
3. PAHO/WHO, Guiding Priciples for Complementary Feeding of the Breastfed Child*.* Division of Health Promotion and Protection, 2003(Washington D.C.).
4. Bruner, J.S. and H. Haste, Making Sense (Routledge Revivals): The Child's Construction of the World. 2010: Routledge.
5. Chang, S.M., et al., Integrating a parenting intervention with routine primary health care: a cluster randomized trial. Pediatrics, 2015;(136): 272-280.
6. Vos, R.C., et al., Developmental trajectories of receptive and expressive communication in children and young adults with cerebral palsy. Dev Med Child Neurol, 2014;(56): 951-959.
7. Hartinger, S.M., et al., Impact of a child stimulation intervention on early child development in rural Peru: a cluster randomised trial using a reciprocal control design. J Epidemiol Comm Health, 2017;(71): 217-224.
8. Wolf, J., et al., Impact of a child stimulation intervention on early child development in rural Peru: a cluster randomised trial using a reciprocal control design. J Epidemiol Community Health, 2017;(71): 217-224.
9. Donnelly, J.E., et al., Physical activity, fitness, cognitive function, and academic achievement in children: a systematic review. Med Sci Sports Exerc, 2016;(48): 1197.
10. Muhoozi, G.K., et al., Nutrition, hygiene, and stimulation education to improve growth, cognitive, language, and motor development among infants in Uganda: A cluster‐randomized trial. Matern Child Nutr, 2018;(14): e12527.
