# Supplementary material for: Cognitive development among children in a low-income setting: Cost-effectiveness analysis of a maternal nutrition education intervention in rural Uganda
Source: PLoS One. 2023 Aug 18;18(8):e0290379. doi: 10.1371/journal.pone.0290379 (PMC10437995; doi:10.1371/journal.pone.0290379)
Supplement: S2 File — (DOCX) [file pone.0290379.s002.docx]

# **S2 Supporting information file**

**Supplementary Methodology for the Economic Evaluation**

Below follows a detailed description of the methods used, to complement the description of methods in the main text.

**Cost identification, quantification, and valuation**

We have followed the pathway on how the nutrition education intervention was delivered to assess health outcome. At the beginning, some costs were identified from the review of the published literature from the RCT [1-3]. Costs were classified according to major expenditure lines to understand the depth of the trial implementation and the associated costs. Table S1 shows the cost items that were included. Overall, most costs were shared across the intervention and control group. The only difference was that the control group did not receive the education intervention. All the capacity building costs were shared equally between the two study groups. Costs varied based on the implementation and monitoring of the education intervention and extra materials used by the intervention group. Unit cost comprised daily participation for training, data collection, and health assessment costs, and these costs were multiplied by the number of days of the involvement and training; while, some personnel costs were on a daily basis (data collectors), and few were on a monthly basis (RCT researchers and volunteers).

**Table S1. Valuation of the intervention costs items**

| **Cost categories** | **Source** | **Type** | **Unit** | **No. of units** | **Unit price** | **Item costs** |
| --- | --- | --- | --- | --- | --- | --- |
|  |  |  |  |  |  |  |
| **Capacity building** |  |  |  |  |  | **1,822** |
| Baseline data collectors training | ^a^ | Wages | 5 Days | 6 | 1.5 | 45 |
| Data collector trainer's | ^a^ | Wages | 5 Days | 1 | 15 | 38 |
| Follow-training | ^a^ | Wages | 6 Days | 4 | 14 | 168 |
| Follow-up trainer | ^a^ | Wages | 6 Days | 1 | 15 | 45 |
| Follow-up trainer | ^a^ | Wages | 6 Days | 3 | 14 | 126 |
| Child development tools -Training | ^a^ | Wages | 6 Days | 4 | 75 | 900 |
| Child development tools -trainer | ^a^ | Wages | 6 Days | 1 | 1,000 | 500 |
| **Personnel** |  |  |  |  |  | **46,485** |
| Leader - Clinical psychologist | ^a,^ | Wages ^c^ | 6-24 months | 1 | 1,607 | 10,847 |
| Leader - Nutritionist | ^a^ | Wages ^c^ | 6-24 months | 1 | 1,607 | 10,847 |
| Baseline anthropometry |  | Wages | 60 days | 12 | 9.50 | 3,420 |
| Baseline psychological tests | ^a^ | Wages | 60 days | 6 | 14 | 2,520 |
| Nutrition education | ^a^ | Wages | 150 sessions | 4 | 14 | 8,400 |
| Adherence to intervention | ^a^ | Wages | Days | 3 | 736.1 | 2,208 |
| Facilitation (via volunteers) | ^a^ | Wages | 18 months | 32 | 5.56 | 3,203 |
| Data collection - (12-16 months) | ^b^ | Wages | 60 Days | 6 | 14 | 2,520 |
| Data collection - (20-24 months) | ^b^ | Wages | 60 Days | 6 | 14 | 2,520 |
| **Materials and others variable cost** |  |  |  |  |  | **11,496** |
| Bayley scales kit (base case) | ^b^ | Real costs | 4 months | 316 | .50 | 830 |
| Bayley scales kit (12-16 months) | ^b^ | Real costs | 4 months | 316 | .50 | 830 |
| Bayley scales kit (20-24 months) | ^b^ | Real costs | 4 months | 316 | .50 | 830 |
| Food demonstration (purchases) | | Real costs | 150 sessions | 150 | 14.50 | 2,175 |
| Toys (pencils, paper) | ^b^ | Real costs | 6-24 months | 316 | 0.69 | 115 |
| Data collection (scales, tapes) | ^b^ | Real costs | 6-24 months | 3.09 | 27.78 | 42 |
| Transportation costs, team^d^ | ^a^ | Rides | 6-24 months | 3 | 961 | 1,442 |
| Car rent for Intervention | ^a^ | Rides | 150 sessions | 150 | 17.50 | 2,625 |
| Transportation costs, mothers^e^ |  | Rides | 12-24 months | 316 | 0.70 | 223 |
| Incentives (T-shirt) | ^a^ | Operation | 6-24 months | 316 | 7.78 | 1,291 |
| Refreshments | ^b^ | Real costs | 12-24 months |  |  | 705 |
| Incentives to mothers | ^b^ | Operation | 12-24months | 316 | 1.17 | 379 |
| **Other overhead/fixed/capital costs** |  |  |  |  |  | **533** |
| Hired special rooms | ^b^ | Real costs | 12-24 months | 316 | 1.39 | 35 |
| Mobile tent | ^a^ | Real costs | 6-24 months | 1 | 850 | 850 |
| Telephone | ^a^ | Real costs | 6-24 months | 3 | 41.67 | 63 |
| **Total cost** |  |  |  |  |  | **60,335** |

The costs were measured in 2014 USD ($).

^a^ Interview with the trial researchers.

^b^ References number 1-3.

^c^ Leaders spent around 3.5 months at baseline, 6 months for both follow-up period.

It was also essential to identify which cost items needed to be included in or excluded from this study. Based on the RCT researchers’ opinion, costs of some items were excluded from this study because they were not relevant for the health outcome of this analysis. Resources used in the intervention were valued according to the market price, and a monetary value assessed for each resource was used for the intervention. Personnel time used, such as for the psychologist, nutritionists, data collector, and mothers, was estimated by combining salaries and benefits.

Table S1 indicates that total intervention costs amounted to $60,335. The majority of resources used were associated with personnel, followed by materials, and then capacity building costs. In the largest cost contributor, personnel cost, the major cost driver was leaders’ costs, which accounted for 46% ($10,847+$10,847). The car rental cost and food demonstration cost accounted for 23% and 19%, respectively, of the materials and other costs. Note that for the comparator (current practice) in the cost effectiveness analysis, we did not include the trial costs for the control group, i.e., we defined the intervention costs relative to the current practice and not relative to the control group.

**Capacity building**

At baseline, 12 data collection personnel participated in a 5-day training session to ensure uniform and standardized procedures. A six-day intensive training (follow-up training) session on the use of the development assessment tool (BSID-III) was conducted by a clinical psychologist to assess child growth and cognitive development. They were four personnel who received six days of training for BSID-III. Only one personnel trainer spent time training those data collectors. They all received a daily wage rate as described in Table S1. All capacity building costs were shared equally between the two study groups because the health personnel divided their time equally between those groups. Table S1 shows the details of all unit costs.

**Personnel cost**

At baseline, for the BSID-III, a trained field-team consisting of psychology graduates independently collected data for 60 days. A village health team leader or a mother leader was selected by consensus in the group to facilitate the intervention. Facilitators conducted a village visit efficiently to help with the intervention process. The health workers also facilitated the follow-up data collection at the age of 12-16 months and 20-24 months, and each follow-up lasted 60 days.

**Table S2. Nutrition education intervention staff**

| **Personnel categories** | **Description** | **Number** | **Salary** |
| --- | --- | --- | --- |
| Leader - clinical psychologist and nutritionist | Management team supporting intervention led by the clinical psychologist | 2 | Monthly |
| Data collection - Baseline | Advanced level of education and Bachelor’s degree graduates | 12 | Part-time |
| Data collection – follow-up | Two nutrition graduates and two child development graduates collected child development data at baseline (6-8 months), and follow-up at 12-16 months and 20-24 months | 4 | Part-time |
| Village health team | Leader or a mother was selected through consensus in the group to facilitate the intervention for 18 months | 32 | Monthly |

Table S2 indicates the number of staff, categories, description, and salary arguments. The clinical psychologist and nutritionist played the most significant role in the implementation of the intervention. Both were offered competitive monthly salary packages. The data collection team comprised of part-time health workers, and they were offered a unit price for their daily participation. All personnel were listed according to their qualifications and time commitments. Qualifications refer to the nature of training, experience and specialized skills required for this intervention.

**Sensitivity analysis**

**Table S3. List of parameters, point estimates in deterministic and probabilistic sensitivity analysis**

| **Input parameter** | **Point estimate**  **(standard error)** | **Uncertainty range^a)^** | **Source of assumption** | **Distribution^b)^** |
| --- | --- | --- | --- | --- |
| **Health outcome, cognitive composite score** | | | | |
| Incremental cognitive composite score^c)^ | 16.11  (2.24) | (11.72-20.50) | Mixed model linear regression;  ref 1, 2^d)^ | Normal |
| **Cost** | | | | |
| Capacity building | 1,822 | (911-2,186) | assumption | Uniform |
| Personnel | 46,485 | (23,243-55,782) | assumption | Uniform |
| Equipment and materials | 11,496 | (9,196-13,794) | assumption | Uniform |
| Capital costs | 533 | (426-640) | assumption | Uniform |

(a) Intervals used in the deterministic sensitivity analyses presented in Figure 2 and Table 3 in the main article. (b) Distribution assumptions used in the probabilistic sensitivity analysis, Figures 3 and 4 in the main text, e.g., “Uniform” indicates that the parameter was assigned a uniform distribution in the probabilistic sensitivity analysis. (c) The incremental cognitive composite score measures the effect of the intervention relative to the control group from baseline to the last follow-up (20-24 months). For this health outcome, the control group in the randomized controlled trial was assumed to represent the current standard of care. (d) We obtained the point estimate and corresponding standard error from a mixed model linear regression. Simple sample means obtained at the last follow-up have previously been reported in the given references.

Table S3 summarize the parameter values and assumptions used in the sensitivity analyses reported in the main article. For instance, the incremental cognitive composite score (health gain) for the intervention groups was 16.11. This point estimate, and its standard error, were obtained from a mixed model regression. In the deterministic sensitivity analysis, we used 11.72 and 20.50 as lower and upper bounds, respectively. In the probabilistic sensitivity analysis, we assumed this input parameter had a normal distribution, for which the mean and standard deviation were set equal to the point estimate and its standard error (16.11 and 2.24, respectively).

**An approximate link between our ICER and the willingness to pay for an avoided Disability Adjusted Life Year (DALY)**

To help guide decision making in low- and middle-income settings, the WHO has suggested to use general willingness to pay thresholds in cost effectiveness analyses. One such willingness to pay threshold per avoided DALY has been set to three times a country’s gross domestic product (GDP) per capita, meaning that interventions that have ICERs (measured in $/DALY) below this threshold are classified as cost-effective. Interventions with ICERs below one GDP per capita per DALY are classified as very cost-effective [4]. In 2014, the low and high thresholds would correspond to 880 $/DALY and 2,640 $/DALY, respectively [5].

In our study, it was not possible to calculate the health consequences in DALYs, because both the DALY weight (the health loss within a single year) and the number of years affected are unknown. However, we will seek to establish an approximate link to the health state “Mild Motor and Cognitive Impairments,” which may have comparable features to our case, and for which the DALY weight has been published [6].

Our results indicated that, on average, a loss of 16.11 units of the cognitive composite score can be avoided at the cost of $265.79 (note that here “loss” means the forgone health gain in the control group, relative to the intervention group). We interpret this as if the willingness to pay threshold relevant for our analysis is $265.79 per avoided case, where one case corresponds to 16.11 units of the cognitive composite score.

Suppose that the unknown DALY weight is such that both our ICER (265.79 $/case) and the 1-GDP-per-capita threshold are satisfied simultaneously. We can then calculate the ratio between our ICER and the one GDP per capita threshold that is equal to 0.3020 DALY/case. If we, for simplicity, ignore discounting and divide that ratio on a period of 60 years, we get 0.005034, which corresponds to the DALY weight per year.

In other words, if the DALY weight is 0.005034 over a 60-year period, then one case corresponds to 0.3020 DALY. Given the willingness to pay threshold of 880 $/DALY, the willingness to pay would be 265.79 $/case. If future studies should reveal that the yet unknown DALY weight is higher than 0.005034, one avoided case would imply more than 0.3020 avoided DALYs and, hence, the willingness to pay would be higher than 265.79 $/case. This would imply that our intervention is very cost effective. When we perform corresponding calculations for the 3-GDP-per-capita threshold (2640 $/DALY), we obtain a DALY weight equal to 0.001678.

**References**

1. Muhoozi GK, Atukunda P, Diep LM, Mwadime R, Kaaya AN, Skaare AB, et al. Nutrition, hygiene, and stimulation education to improve growth, cognitive, language, and motor development among infants in Uganda: A cluster‐randomized trial. Maternal & Child Nutrition. 2018;14(2): e12527.

2. Muhoozi GK, Atukunda P, Mwadime R, Iversen PO, Westerberg AC. Nutritional and developmental status among 6-to 8-month-old children in southwestern Uganda: a cross-sectional study. Food & Nutrition Research. 2016;60(1): 30270.

3. Atukunda P, Muhoozi GK, Westerberg AC, Iversen PO. Nutrition, hygiene and stimulation education for impoverished mothers in rural Uganda: effect on maternal depression symptoms and their associations to child development outcomes. Nutrients. 2019;11(7): 1561.

4. Marseille E, Larson B, Kazi DS, Kahn JG, Rosen S. Thresholds for the cost-effectiveness of interventions: alternative approaches. Bull World Health Organ. 2015;93:118–124.

5. World Development Indicators [Internet]. 2015 [Accessed 22.10.2022]. Available from:<https://data.worldbank.org/indicator/NY.GDP.PCAP.CD?end=2015&locations=UG&start=2010&view=chart>.

6. Salomon JA, Haagsma JA, Davis A, Noordhout CM, Polinder S, Havelaar AH, Cassini A, Devleesschauwer B, Kretzschmar M, N Speybroeck, Murray CJL, Vos T. Disability weights for the Global Burden of Disease 2013 Study. Lancet Glob Health 2015;3: e712-e23.
